# Structural and functional characterization of a novel cold-active *S*-formylglutathione hydrolase (*Sf*SFGH) homolog from *Shewanella frigidimarina,* a psychrophilic bacterium

**DOI:** 10.1186/s12934-019-1190-1

**Published:** 2019-08-19

**Authors:** Chang Woo Lee, Wanki Yoo, Sun-Ha Park, Ly Thi Huong Luu Le, Chang-Sook Jeong, Bum Han Ryu, Seung Chul Shin, Han-Woo Kim, Hyun Park, Kyeong Kyu Kim, T. Doohun Kim, Jun Hyuck Lee

**Affiliations:** 1 Unit of Polar Genomics, Korea Polar Research Institute, Incheon, 21990 Republic of Korea; 2 Department of Chemistry, College of Natural Science, Sookmyung Woman’s University, Seoul, 04310 Republic of Korea; 3 Department of Molecular Cell Biology, Samsung Biomedical Research Institute, Sungkyunkwan University School of Medicine, Suwon, 16419 Republic of Korea; 4 Department of Polar Sciences, University of Science and Technology, Incheon, 21990 Republic of Korea; 5 Division of Biotechnology, College of Life Sciences and Biotechnology, Korea University, Seoul, 02841 Republic of Korea; 6 Present Address: Public CDMO for Microbial-based Vaccine, Infrastructure Project Organization for Global Industrialization of Vaccine, Hwasun, 58141 Republic of Korea

**Keywords:** Crystal structure, *S*-Formylglutathione hydrolase, Substrate specificity, *Shewanella frigidimarina*, Mutagenesis

## Abstract

**Background:**

*S*-Formylglutathione is hydrolyzed to glutathione and formate by an *S*-formylglutathione hydrolase (SFGH) (3.1.2.12). This thiol esterase belongs to the esterase family and is also known as esterase D. SFGHs contain highly conserved active residues of Ser-Asp-His as a catalytic triad at the active site. Characterization and investigation of SFGH from Antarctic organisms at the molecular level is needed for industrial use through protein engineering.

**Results:**

A novel cold-active *S*-formylglutathione hydrolase (*Sf*SFGH) from *Shewanella frigidimarina*, composed of 279 amino acids with a molecular mass of ~ 31.0 kDa, was characterized. Sequence analysis of *Sf*SFGH revealed a conserved pentapeptide of G-X-S-X-G found in various lipolytic enzymes along with a putative catalytic triad of Ser148-Asp224-His257. Activity analysis showed that *Sf*SFGH was active towards short-chain esters, such as *p*-nitrophenyl acetate, butyrate, hexanoate, and octanoate. The optimum pH for enzymatic activity was slightly alkaline (pH 8.0). To investigate the active site configuration of *Sf*SFGH, we determined the crystal structure of *Sf*SFGH at 2.32 Å resolution. Structural analysis shows that a Trp182 residue is located at the active site entrance, allowing it to act as a gatekeeper residue to control substrate binding to *Sf*SFGH. Moreover, *Sf*SFGH displayed more than 50% of its initial activity in the presence of various chemicals, including 30% EtOH, 1% Triton X-100, 1% SDS, and 5 M urea.

**Conclusions:**

Mutation of Trp182 to Ala allowed *Sf*SFGH to accommodate a longer chain of substrates. It is thought that the W182A mutation increases the substrate-binding pocket and decreases the steric effect for larger substrates in *Sf*SFGH. Consequently, the W182A mutant has a broader substrate specificity compared to wild-type *Sf*SFGH. Taken together, this study provides useful structure–function data of a SFGH family member and may inform protein engineering strategies for industrial applications of *Sf*SFGH.

**Electronic supplementary material:**

The online version of this article (10.1186/s12934-019-1190-1) contains supplementary material, which is available to authorized users.

## Background

Esterases hydrolyze ester bonds and produce cleaved acid and alcohol compounds. Although they have low sequence identity and diverse substrate specificities as well as biological functions, most esterases adopt a common α/β hydrolase fold and contain a highly conserved serine–histidine–aspartic acid catalytic triad at the active site [1–3]. *S*-formylglutathione hydrolase (SFGH, EC 3.1.2.12) is a member of the esterase family and is also known as esterase D. SFGH hydrolyzes *S*-formylglutathione into glutathione and formic acid and can be broadly considered an esterase, or more specifically, a thioesterase. This enzyme is involved in the glutathione-dependent formaldehyde detoxification pathway, which prevents the negative effects of formaldehyde by a series of enzymatic reactions involving formaldehyde dehydrogenase (FALDH, *S*-hydroxymethylglutathione dehydrogenase) and SFGH, which depend upon the spontaneous binding of glutathione to formaldehyde. Indeed, the expression levels of FALDH (*frmA*) and SFGH (*frmB*) in *Escherichia coli* cells are remarkably increased by 20- to 100-fold over basal levels when subjected to formaldehyde treatment [4]. Notably, a double-mutant strain of *E. coli* with the deletion of both *frmB* and paralogous *yeiG* genes, which all encode SFGH, showed a remarkably reduced growth rate in the presence of formaldehyde [5]. Likewise, a mutant strain of *Paracoccus denitrificans* for the SFGH gene (*fghA*) was unable to grow on methanol or methylamine, which resulted in the formation of formaldehyde [6]. Considering the importance of this pathway, SFGH genes are widely conserved in diverse organisms, including prokaryotes and eukaryotes. In humans, esterase D, which was subsequently proven to be identical to SFGH, has been identified, characterized, and structurally determined. This enzyme has attracted much attention as its polymorphisms are related to several diseases, including retinoblastoma and Wilson’s disease [7–9]. In addition, SFGHs were found in other eukaryotes including plants (*Arabidopsis thaliana* [10, 11] and *Pisum sativum* [12]) and yeast (e.g., *Saccharomyces cerevisiae* [13] and *Candida boidinii* [14]). Bacterial SFGHs were also identified in various species including *Paracoccus denitrificans* [6], *Agrobacterium tumefaciens* [15], and *E. coli* [5].

As mentioned above, many studies on SFGHs have been conducted, which have revealed the features of SFGHs. Sequence alignment analyses have shown that SFGH has a conserved sequence motif (GHSMGG) that harbors catalytic serine; thus, this enzyme is also referred to as a serine hydrolase. This serine residue, along with the conserved aspartate and histidine residues, forms a catalytic triad of SFGH. Two conserved residues of leucine and methionine that form an oxyanion hole are also found in close proximity to the catalytic serine. Furthermore, enzymes belonging to the SFGH class generally contain five large blocks of conserved residues [5]. Functional characterization studies demonstrated that SFGH could also exhibit carboxyl esterase activity towards xenobiotic esters (e.g., methylumbelliferyl acetate) in addition to having thioesterase activity [10, 11, 13]. Many SFGHs are known to be active towards *p*-nitrophenyl esters or naphthyl ester derivatives as well as formyl glutathione.

In this study, we characterized a novel cold-active *S*-formylglutathione hydrolase (*Sf*SFGH) homolog from a psychrophilic bacterium, *Shewanella frigidimarina*. *Shewanella frigidimarina* is a facultative anaerobic Gram-negative bacterium with rod-like shape, originally isolated from Antarctic marine environments. To date, only two studies have characterized SFGH from Arctic/Antarctic organisms: OLEI01171 from *Oleispira antarctica* [16] and *Ph*EST from *Pseudoalteromonas haloplanktis* [17, 18]. However, the structural and functional properties of *S*-formylglutathione hydrolase from psychrophiles are still poorly understood. In this context, our study will enrich the pool of SFGH, providing useful information regarding the sequence, biophysical, and enzymatic characteristics of SFGH. Furthermore, the crystal structure of *Sf*SFGH has been determined at 2.32 Å resolution. Several substrate-binding site residue mutants have been generated to alter the substrate specificity and flexibility of *Sf*SFGH. Interestingly, the W182A mutant showed remarkably increased substrate flexibility compared to wild-type *Sf*SFGH. Collectively, these findings provide useful insights to inform the protein engineering of bacterial esterases as industrially useful biocatalysts.

## Results and discussion

### Biochemical characterization of *Sf*SFGH

The sequence of *Sf*SFGH (GenBank I.D.: ABI73260.1) was previously annotated as a carboxylesterase with little additional information. Sequence alignment of *Sf*SFGH with similar sequences retrieved from PSI-BLAST indicates that these enzymes share highly conserved sequences, including a ‘GHSMGG’ motif. These enzymes are now classified as a PF00756 esterase family based on sequence homology [15, 19–21]. As the ‘GHSMGG’ motif is a representative feature of the esterase/lipase family V, a phylogenetic tree was generated containing *Sf*SFGH with other enzymes belonging to the esterase/lipase family from I to VIII. As expected, *Sf*SFGH was classified into the esterase/lipase family V and showed a clear separation from other families (Additional file 1: Fig. S1). Furthermore, it is generally known that the ‘PAL’ motif is one of the representative features of the esterase/lipase family V [22]. Together with *Sf*SFGH, other PF00756 esterases, such as OLEI01171 and *Ph*Est, have the ‘PAL’ and ‘PML’ motifs, respectively. Collectively, sequence analysis data suggest that *Sf*SFGH might be a new member of the esterase/lipase family V. In addition, *Sf*SFGH shows significant sequence homology with other bacterial SFGHs (66.43% sequence identity with *Pseudoalteromonas haloplanktis* TAC125 SFGH, 54.48% with *Paracoccus denitrificans* SFGH, 50.91% with *Agrobacterium tumefaciens* SFGH, and 67.93% with *E. coli* SFGH). We also performed gene cluster analysis of *Sf*SFGH, and found that gene clusters surrounding the SFGH gene were highly conserved in *Shewanella* species, including *S. sediminis*, *S. woodyi*, *S. pealeana*, and *S. piezotolerans* (Additional file 1: Fig. S2).

**Fig. 1:**
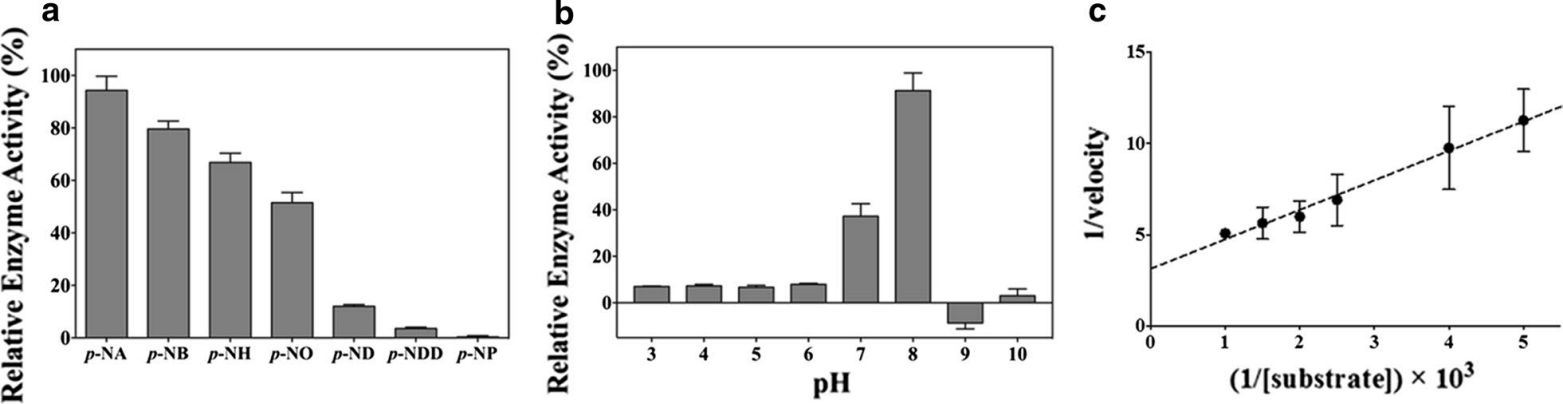
Enzymatic activity of *Sf*SFGH. **a** Substrate specificity was investigated using *p*-nitrophenyl esters with different acyl-chain lengths. **b** Effects of pH on enzymatic activity were studied from pH 3.0 to 10.0. Activity at the optimal pH was set as 100%. **c** Lineweaver–Burk plots showing the reciprocal of the velocity of *Sf*SFGH versus the reciprocal of the substrate concentration. All experiments were performed in triplicate

**Fig. 2:**
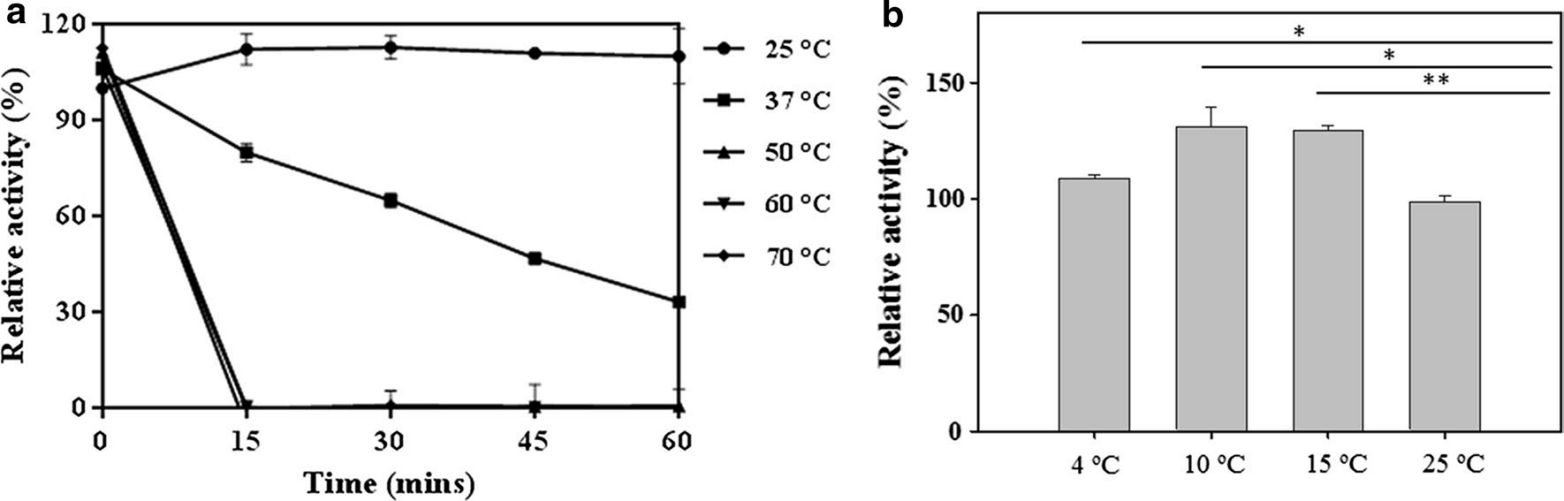
Effects of temperature on the activity of *Sf*SFGH. **a** Thermal stability of *Sf*SFGH. *Sf*SFGH was heated at various temperatures, ranging from 25 to 70 °C. Then, 10 μg *Sf*SFGH was collected every 15 min and the residual activity was measured using *p*-NA as a substrate. Activity is shown relative to the initial activity. **b** Enzymatic activity of *Sf*SFGH was evaluated at different temperatures. Relative activity of *Sf*SFGH was measured using *p*NP-C_2_ as a substrate after 1 h incubation at each temperature. A two-tailed unpaired Student *t* test was used to evaluate statistical significance: *P < 0.05 and **P < 0.005. All experiments were performed in triplicate

Recombinant *Sf*SFGH protein was expressed in *E. coli* and purified by His-tag affinity chromatography and size-exclusion chromatography (Additional file 1: Fig. S3). Analytical ultracentrifugation (AUC) analysis with *Sf*SFGH showed a dimer mass of 62.3 kDa (sedimentation coefficient, 4.209 S; frictional ratio, 1.34). The substrate specificity of recombinant *Sf*SFGH protein was determined using *p*-nitrophenyl esters with different acyl-chain lengths. As shown in Fig. 1a, *Sf*SFGH showed a preference toward short-chain substrates, including *p*-NA, *p*-NB, *p*-NH, and *p*-NH, with more than 50% of its maximum activity. However, less than 15% of relative enzymatic activities were detected towards longer-chain substrates, such as *p*-ND and *p*-NDD, and almost no enzymatic activity was observed with *p*-NP. The optimum pH for *Sf*SFGH was determined to be in the range of pH 3.0 to 10.0 (Fig. 1b). *Sf*SFGH showed maximum activity at pH 8.0 and ~ 40% activity at pH 7.0. In contrast, *Sf*SFGH exhibited almost no enzymatic activity in other pH ranges. The kinetic parameters of *Sf*SFGH were determined using *p*-NA. *Sf*SFGH exhibited typical Michaelis–Menten kinetics with a hyperbolic plot of reaction velocity as a function of substrate concentration. Based on the Lineweaver–Burk plot with linear regression, the Michaelis–Menten constant (*K*_m_) and maximum velocity (*V*_max_) were 515 µM (± 42.8) and 0.319 µM s^−1^ (± 0.0254), respectively (Fig. 1c). The calculated *k*_cat_ of *Sf*SFGH was 0.310 s^−1^ (± 0.0258), which is almost six times less than that of *Atu*SFGH and *Ph*EST (1.95 s^−1^ and 2 s^−1^, respectively) [15, 17]. Notably, enzymatic activity assay results for several different temperatures showed that *Sf*SFGH has high activity at low temperature (4 °C), but starts to lose activity at ~ 37 °C (Fig. 2).

### Overall structure of *Sf*SFGH

**Fig. 3:**
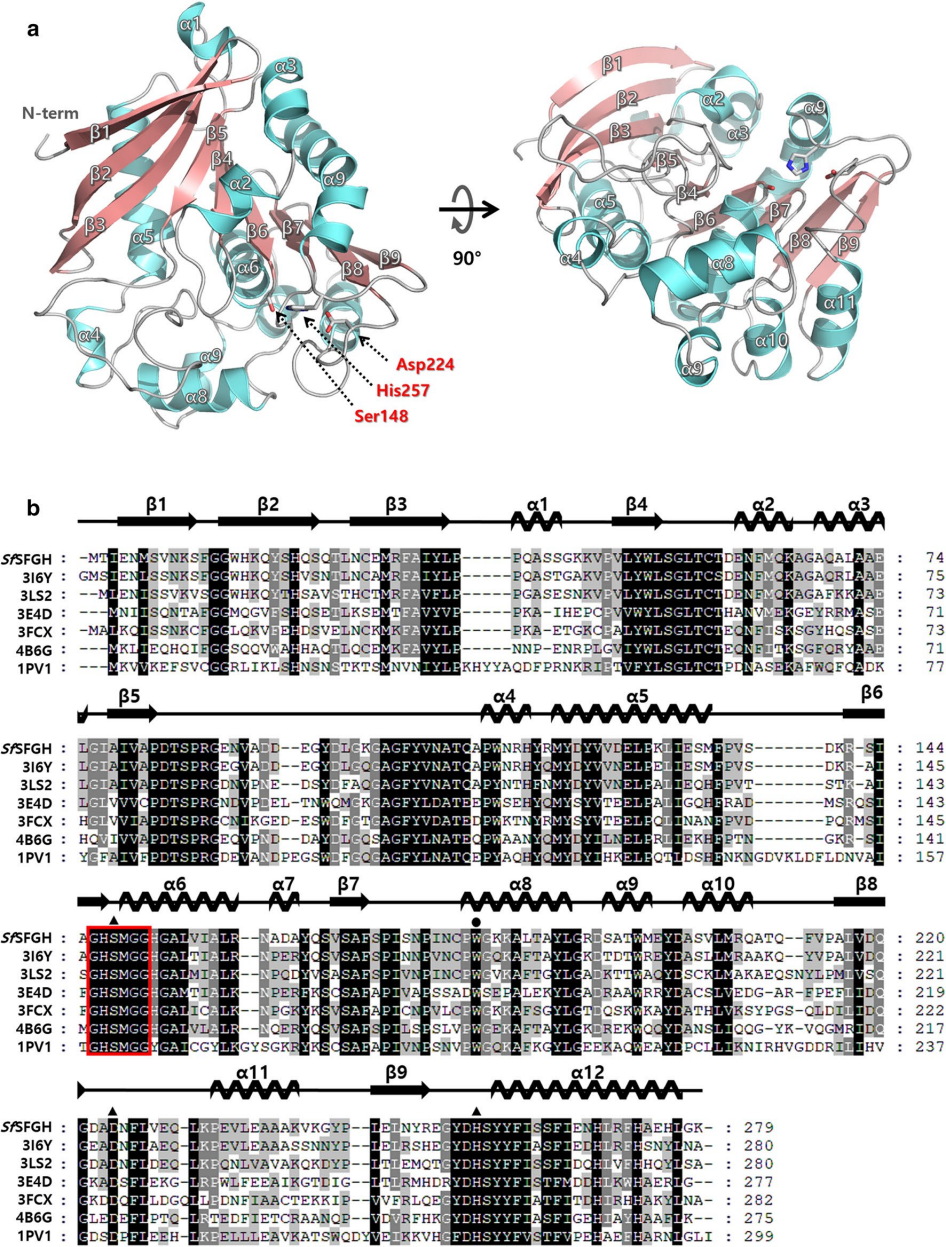
Crystal structure of *Sf*SFGH. **a** The overall structure of *Sf*SFGH is shown as a ribbon diagram with α-helices (aquamarine color) and β-strands (salmon color). The catalytic triad residues (Ser148, Asp24, and His257) are shown as stick models. **b** Multiple sequence alignments of *Sf*SFGH (NCBI reference sequence number WP_011638859.1) with homologous models. The sequences of esterases from the oil-degrading bacterium *Oleispira antarctica* (PDB code 3i6y; UniProtKB code D0VWZ4), *S*-formylglutathione hydrolase from *Pseudoalteromonas haloplanktis* (PDB code 3ls2; UniProtKB code Q3IL66), *S*-formylglutathione hydrolase from *Agrobacterium tumefaciens* (PDB code 3e4d; UniProtKB code A9CJ11), esterase D from humans (PDB code 3fcx; UniProtKB code P10768), esterase D from *Neisseria meningitides* (PDB code 4b6g; UniProtKB code Q9JZ43), and *S*-formylglutathione hydrolase from *Saccharomyces cerevisiae* (PDB code 1pv1; UniProtKB code P40363) were used for alignment. The conserved motif found in SFGHs is boxed in red. The catalytic triad residues indicated with a black triangle are conserved in all models. The gatekeeper residue of tryptophan is indicated with a black circle. An alignment was prepared using the program *ClustalX* and edited with *GeneDoc*. Corresponding secondary structures are shown above the sequences

The crystal structure of *Sf*SFGH was determined to a resolution of 2.32 Å using a molecular replacement method (Fig. 3). The crystal structure of esterase (OLEI01171) from *Oleispira antarctica* (sequence identities 83%; PDB cod 3i6y) was used as a template model [16]. *Sf*SFGH was crystallized in space group *P*2_1_ with two monomers in the asymmetric unit. The final model contained 208 water molecules and 554 amino acids. The monomeric structure of *Sf*SFGH includes 12 α-helices and 9 β-strands (Fig. 3a). The β1–β2–β3 strands form an antiparallel β-sheet, and the β4 to β9 strands form a parallel β-sheet. These two β-sheets are linked and assemble into a large central β-sheet. The 12 α-helices surround both sides of the large β-sheet. These features of the overall architecture show considerable similarity with those of the previously reported *S*-formylglutathione hydrolases (SFGHs). Structural alignments using a Dali search showed that the esterase (OLEI01171) from *Oleispira Antarctica* (PDB code 3i6y) has the highest Z-score with 50.5 [16, 23]. Crystal structures of *S*-formylglutathione hydrolase from *Pseudoalteromonas haloplanktis* (PDB code 3ls2), esterase D from *Neisseria meningitidis* (PDB code 4b6g), and *S*-formylglutathione hydrolase from *Agrobacterium fabrum* (PDB code 3e4d) also showed high structural similarities (Table 1) [15, 18, 24].

**Table 1:** Selected structural homologues of *Sf*SFGH from a DALI search (DALI-Lite server)

Protein	PDB code	DALI Z-score	R.m.s.d. (Å)	Sequence % ID with *Sf*SFGH (aligned residue number/total residue number)	References
Esterase APC40077	3I6Y	50.7	0.4	81% (277/278)	[16]
*S*-Formylglutathione hydrolase	3LS2	48.8	0.8	66% (276/278)	[18]
Putative esterase	4B6G	46.8	0.9	53% (275/275)	[24]
Esterase D	3E4D	46.4	1.0	48% (276/278)	[15]
*S*-Formylglutathione hydrolase	3FCX	46.0	1.0	54% (271/275)	[8]
Hypothetical 33.9 kDa Esterase in SMC3-MRPL8	1PV1	42.3	1.2	45% (272/290)	[25]
*S*-Formylglutathione hydrolase	4FLM	42.1	1.1	44% (269/288)	[38]

### Active site of *Sf*SFGH

**Fig. 4:**
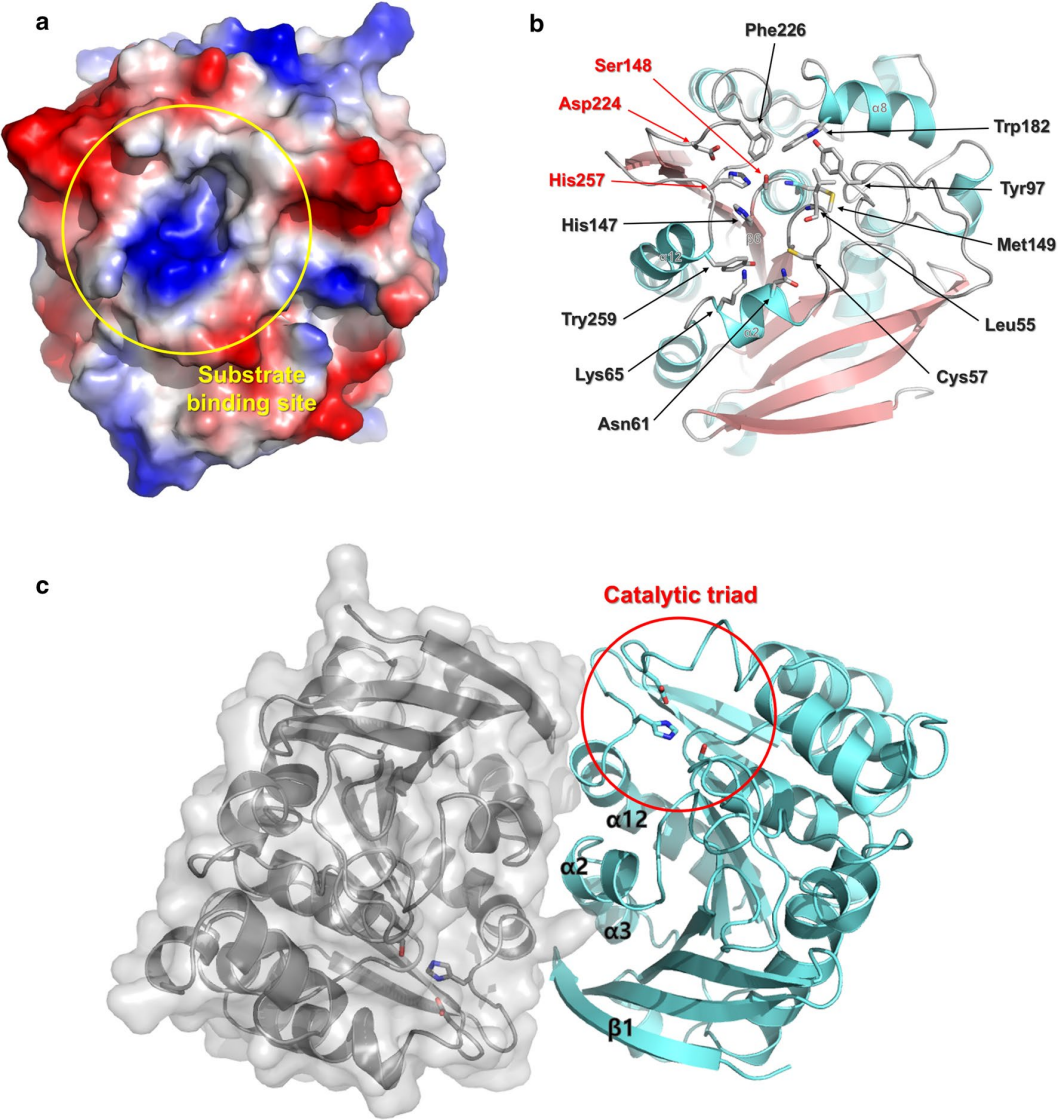
Active site of *Sf*SFGH. **a** The substrate-binding site (yellow circle) of *Sf*SFGH has a positively charged surface. **b** The conserved catalytic triad (Ser148, Asp24, and His257) residues are located on the substrate-binding site. The residues creating a substrate-binding site are presented as a stick model. **c** Dimerization of *Sf*SFGH is mediated by the α2, α3, and α12 helices. The β1-strand also participates in the dimerization interaction

The putative substrate-binding site of *Sf*SFGH is located at the end of the β6-strand. Including the α2, α8, and α12 helices, the β4-α2 loop, β8-α11 loop, and β9-α12 loop regions together form a groove architecture. The conserved catalytic triad residues of Ser148, Asp224, and His257 are located in this groove (Fig. 4). The catalytic residue Ser148 is located in the short β6-α6 loop region and directly interacts with His257 located in the β9-α12 loop region. Similarly, the residue Asp224 located on the β8-α11 loop region interacts with His257. In addition, the oxyanion hole comprising the backbone amides Leu55 and Met149 can be observed at the neighboring active site. In the apo state of the *Sf*SFGH structure, oxyanion holes are occupied by water molecules. The vicinity of the substrate-binding site is positively charged by the Lys65 and His147. This positively charged area might induce binding of an acidic substrate, such as an acyl group. Moreover, the residues Cys57, Asn61, Trp182, and Phe226 are conserved in the substrate-binding site of *Sf*SFGH. Especially in SFGH from *Saccharomyces cerevisiae*, the W197I mutant (Trp182 of *Sf*SFGH) showed highly increased substrate affinities [25]. Likewise, mutation of Trp182 to alanine in *Sf*SFGH affected its activity. Detailed data for the W182A mutant are provided in the next section.

### Site-directed mutagenesis to increase the substrate-binding pocket size of *Sf*SFGH

**Fig. 5:**
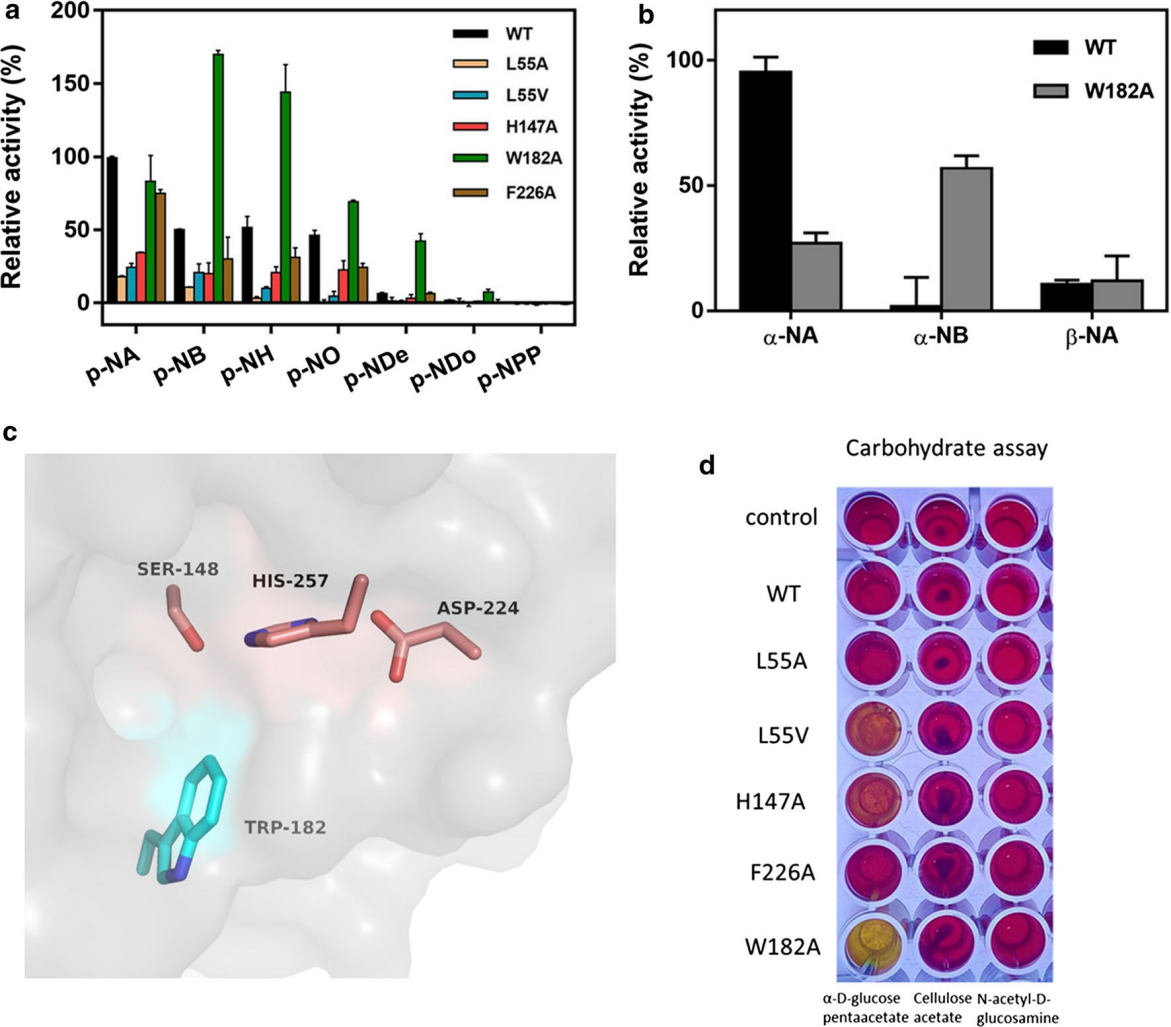
Effects of mutation on *Sf*SFGH enzymatic activity. **a** Comparison of relative activities between wild-type *Sf*SFGH and several point mutants (L55A, L55 V, H147A, W182A, and F226A). All mutants are located on the substrate-binding pocket. Assays were carried out using various substrates (*p*-NA, *p*-NB, *p*-NH, *p*-NO, *p*-NDe, *p*-NDo, and *p*-NPP). The relative activity of wild-type *Sf*SFGH with *p*-NA was set to 100%. **b** Increased enzymatic activity for α-NB in the W182A mutant was observed compared with that of wild-type *Sf*SFGH. The relative activity of wild-type *Sf*SFGH with α-NA was set to 100%. **c** The surface of the substrate-binding site of *Sf*SFGH is colored grey. The catalytic triad residues (Ser148, Asp224, and His257; salmon) and Trp182 (cyan) are presented as a stick model. **d** Hydrolytic activities towards α-d-glucose penta-acetate, cellulose acetate, and *N*-acetyl-d-glucosamine. All experiments were performed in triplicate

Based on this structural information, we hypothesized that remodeling the substrate-binding pockets of *Sf*SFGH would allow the enzyme to exhibit broadened substrate specificity. Therefore, several mutants targeting the substrate-binding pocket residues (L55A, L55V, H147A, W182A, and F226A) were designed, constructed, purified, and characterized with respect to their activity (Fig. 5a). Interestingly, the W182A mutant showed substantially increased activity for large and bulky substrates compared to wild-type *Sf*SFGH. Notably, the W182A mutant showed a substantially altered preference for longer substrates (*p*-NB and *p*-NH), unlike wild-type *Sf*SFGH (Fig. 5a, b). Likewise, the *K*_*m*_ values of W182A for *p*-NA, *p*-NB, and *p*-NO were 1.393 (± 0.136), 0.303 (± 0.049), and 0.459 (± 0.085), respectively. The calculated catalytic efficiency (*k*_*cat*_/*K*_*m*_) of W182A for *p*-NB (26.4 s^−1^ mM^−1^) was 20 times greater than that for *p*-NA (1.3 s^−1^ mM^−1^) (Table 2). In addition, the W182A mutant also showed acetylation activity towards α-d-glucose penta-acetate whereas the wild-type did not. It is thought that the W182A mutation probably eliminates the steric hindrance of substrate entry and increases the size of the substrate-binding pocket by removing the bulky side chain of Trp182 (Fig. 5c, d). However, other mutations were not effective for changing the substrate specificity and improving the activity of *Sf*SFGH. Therefore, the Trp182 residue is important for substrate selectivity and discrimination in *Sf*SFGH. In conclusion, the W182A mutant of *Sf*SFGH has different substrate specificities and unique biochemical characteristics. Thus, the engineered W182A mutant may be useful for biotechnological fields, such as fine chemical synthesis and industrial pharmaceutics.

**Table 2:** *K*_m_, *k*_cat_, and specificity constant (*k*_cat_/*K*_m_) of the W182A mutant

Substrates	V_max_ (μM s^−1^)	*K*_*m*_ (mM)	*k*_*cat*_ (S^−1^)	*k*_*cat*_/*K*_*m*_ (s^−1^ mM^−1^)
*p*-NA	5.378 ± 0.268	1.393 ± 0.136	1.852 ± 0.092	1.3
*p*-NB	0.861 ± 0.035	0.303 ± 0.049	8.003 ± 0.326	26.4
*p*-NO	0.477 ± 0.023	0.495 ± 0.085	0.144 ± 0.007	0.3

### Structural stability of *Sf*SFGH

**Fig. 6:**
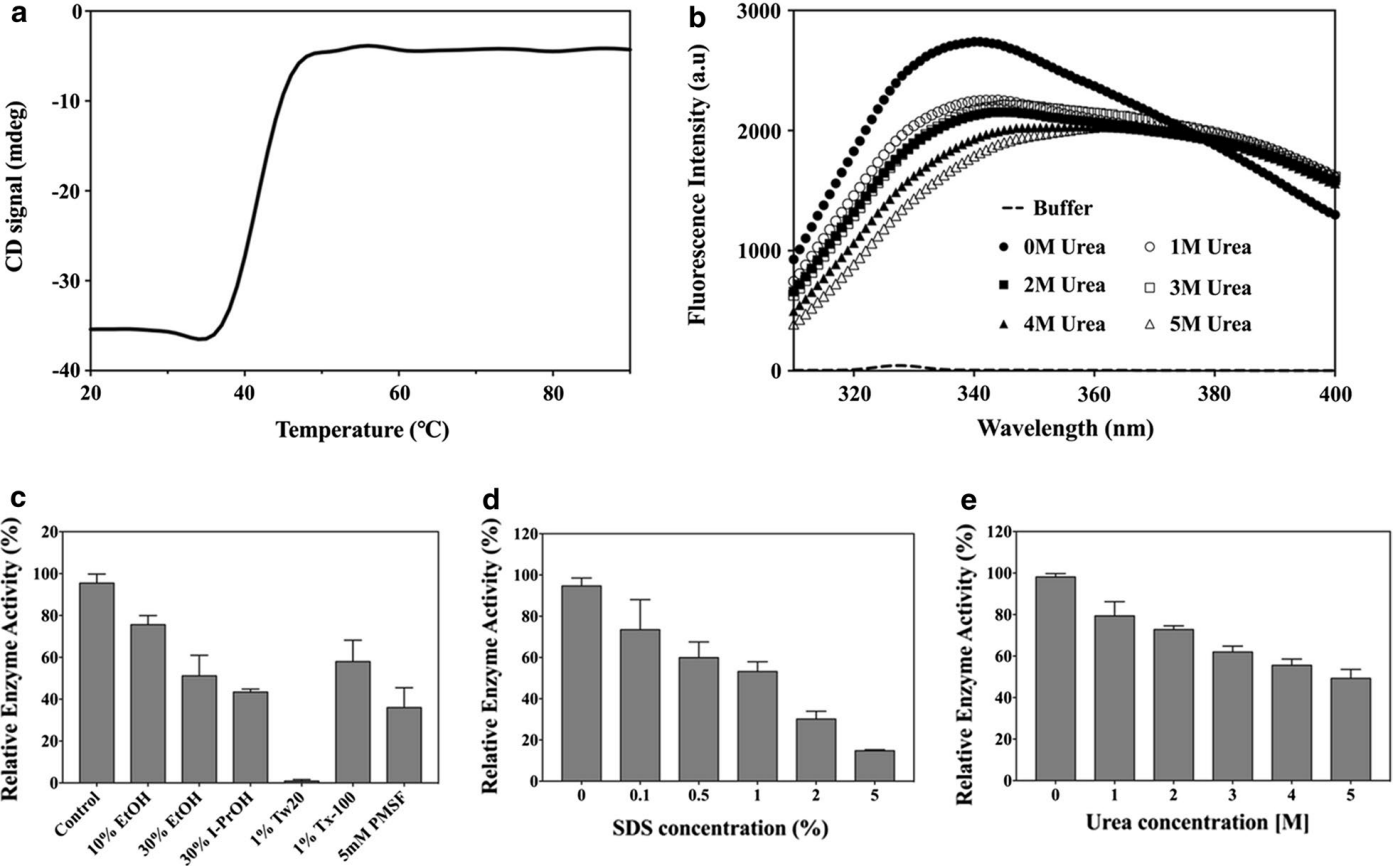
Stability of *Sf*SFGH. **a** Thermal unfolding of *Sf*SFGH was monitored at 222 nm from 20 to 90 °C using far-UV CD measurements. **b** Intrinsic fluorescence spectra were recorded with increasing concentrations of urea from 0 to 5 M. **c** Chemical stability of *Sf*SFGH. Residual activities were measured after incubation of the enzyme for 1 h in various conditions. Effects of SDS (**d**) and urea (**e**) on the enzymatic activity of *Sf*SFGH. All experiments were performed in triplicate

The structural stability of *Sf*SFGH was investigated via thermal and chemical unfolding with circular dichroism and intrinsic fluorescence analyses, respectively. For thermal unfolding, CD signals of *Sf*SFGH were monitored at 222 nm, at temperatures from 20 to 90 °C. In these Far-UV CD measurements, *Sf*SFGH showed a remarkable structural transition at around 45 °C (Fig. 6a), which is consistent with the results of the enzymatic activity assays (Fig. 2a). In the case of OLEI01171, another cold-active esterase, enzymatic activity decreased when the temperature increased, and the aggregation temperature is similar to that of *Sf*SFGH (~ 45 °C) [16]. Structural superposition analysis shows how *Sf*SFGH remains catalytically active at low temperatures. *Sf*SFGH has a more open active site compared with mesophilic homologs (human esterase D, PDB code 3FCX, and *Saccharomyces cerevisiae* SFGH, PDB code 4FLM) [9, 38]. Especially, the β5-α4 loop region (residues 83–112) in *Sf*SFGH has a significantly different conformation with a relatively higher main chain B-factor value (27.9 Å^2^) than overall B-factor value (24.7 Å^2^) (Additional file 1: Fig. S4). Our sequence alignment results also show that only cold-active enzymes have a conserved glycine residue in this β5-α4 loop region (Gly96 in *Sf*SFGH and Gly96 in OLEI01171). Collectively, it is thought that the flexibility of the β5-α4 loop region and the open active site are related to the cold-activity of *Sf*SFGH.

Next, chemical unfolding of *Sf*SFGH was monitored based on changes in intrinsic fluorescence with different urea concentrations (from 0 to 5 M). As shown in Fig. 6b, native *Sf*SFGH exhibited a λ_max_ of 341 nm in the absence of urea, and a red shift of λ_max_ to 367 nm was observed in the presence of 5 M urea. Fluorescence intensity was significantly decreased from 1 M urea, and further reduction was nearly proportional to the increase in urea concentration.

In the chemical stability test, *Sf*SFGH showed remarkable residual enzymatic activity in the presence of diverse chemicals (Fig. 6c–e). With organic solvents, *Sf*SFGH could retain ~ 76%, ~ 51%, and ~ 43% of its original activity with 10% EtOH, 30% EtOH, and 30% I-PrOH, respectively. For detergents, *Sf*SFGH exhibited more than 50% of its initial activity with 1% Triton X-100 or SDS up to 1%, although almost no enzymatic activity was observed with 1% Tween-20. Higher SDS concentrations of 2% and 5% decreased the enzymatic activity to 30% and 15% of its original activity, respectively, rather than inducing complete inhibition of enzymatic activity. Notably, *Sf*SFGH incubated with 5 mM PMSF, a serine hydrolase inhibitor, retained ~ 36% of its original activity. To verify the resistance of *Sf*SFGH to hydrogen-bond-disturbing conditions, *Sf*SFGH activity was also examined with urea concentrations ranging from 0 to 5 M. The enzymatic activity of *Sf*SFGH was found to be gradually reduced as the concentration of urea increased. Interestingly, *Sf*SFGH retained more than ~ 50% of its initial activity at all concentrations. Furthermore, *Sf*SFGH maintained its activity (≥ 80%) even after 10 repeated cycles of freezing and thawing (Additional file 1: Fig. S5). Taken together, *Sf*SFGH appears to have notable thermal and chemical stability. Notably, *Sf*SFGH was strongly resistant to organic solvents and detergents such as EtOH, i-PrOH, Triton X-100, and SDS, suggesting that it could be useful in industrial applications.

## Conclusion

In this study, a novel cold-active *S*-formylglutathione hydrolase (*Sf*SFGH) homolog from *Shewanella frigidimarina* was characterized. Regarding enzymatic activity, *Sf*SFGH was found to prefer short-chain substrates and weakly basic pH conditions. A single residue mutation (W182A) changed the substrate preference of *Sf*SFGH to longer-chain substrates. This finding provides valuable information for further protein engineering of *Sf*SFGH to obtain more suitable substrate-binding sites. Remarkably, *Sf*SFGH appears to retain its enzymatic activity in the presence of inhibitory chemicals. These properties of *Sf*SFGH could be very useful in industrial applications that generally require enzymes to show robust activity in harsh conditions.

## Materials and methods

### Protein expression and purification

*Shewanella frigidimarina* (KCTC 22881, Korean Collection for Type Cultures (WDCM 597), Seoul, Korea) was cultured in marine medium 2216 (BD Difco, USA). Chromosomal DNA was extracted and purified using a DNeasy Tissue and Blood Kit (Qiagen, USA) according to the manufacturer’s instructions. The open reading frame of the *Sf*SFGH gene was amplified using polymerase chain reaction with the following primers: forward, 5′-CCTGCTAGCATGACAATTGAAAATATGAG-3′; reverse, 5′-CTAACTCGAGCTTTCCCAGATGCTC-3′. The PCR product was inserted into a pET21a vector, and pET21a-*Sf*SFGH was cloned into the *E. coli* BL21(DE3) stain. Transformed cells were grown in LB medium containing 100 μg mL^−1^ ampicillin at 37 °C until the OD600 reached 0.5. Then, isopropyl-β-d-1-thiogalactoside (IPTG) was added to a final concentration of 1 mM and the cells were incubated for 3 h to overexpress *Sf*SFGH. The cells were harvested by centrifugation at 5000 rpm for 20 min at 4 °C. The resulting pellet was then resuspended in binding buffer (20 mM Tris–HCl, 300 mM NaCl, 20 mM imidazole, pH 8.0), followed by sonication. The lysate was centrifuged at 40,000×*g* for 10 min, and the supernatant was applied to a 1 mL HisTrap FF column under the AKTA Start system (GE Healthcare). After an extensive washing step with binding buffer, bound *Sf*SFGH was eluted with elution buffer (20 mM Tris–HCl, 300 mM NaCl, 250 mM imidazole, pH 8.0). The purified *Sf*SFGH was desalted using a PD-10 column (GE Healthcare) with an assay buffer (20 mM Tris–HCl, 200 mM NaCl, pH 8.0). The molecular mass of *Sf*SFGH in the native state was estimated using size exclusion chromatography with a Sephacryl S200 HR column (GE healthcare) in an AKTA Start FPLC system (GE healthcare).

### Crystallization and data collection of *Sf*SFGH

Initial crystallization screening of *Sf*SFGH was performed using the sitting-drop vapor-diffusion method at 293 K in 96-well crystallization plates (Emerald Bio, Bainbridge Island, WA, USA) using a Mosquito crystallization robot system (TTP Labtech, Cambridge, MA, USA) with commercially available screening kits (MCSG I-IV; Microlytic Burlington, MA, USA). Crystallization drops consisting of 300 nL protein solution mixed with 300 nL precipitant solution were equilibrated against 80 µL reservoir solution. Crystals of *Sf*SFGH appeared in the condition of 0.2 M lithium chloride and 20% (w/v) PEG 3350 (MCSG II #A11). Further optimization of crystallization was conducted in 24-well plates using the hanging-drop vapor-diffusion method by varying the salt and PEG concentrations. Optimized crystals of *Sf*SFGH were obtained from the condition of 0.2 M lithium chloride and 16% (w/v) PEG 3350. Before mounting, a single crystal of *Sf*SFGH was briefly soaked in Paratone-N oil (Hampton Research, Aliso Viejo, USA) for cryoprotection from the nitrogen-gas stream (100 K). Diffraction data were collected on a beamline BL-5C at the Pohang Accelerator Laboratory (PAL; Pohang, Korea) using X-rays at a wavelength of 0.9796 Å. The crystal was oscillated by 1° after every 1 s exposure. Data containing 200 images were processed and scaled using *HKL*-2000 [26]. Data collection and processing statistics are shown in Table 3.

**Table 3:** X-ray diffraction data collection and refinement statistics

Data set	*Sf*SFGH
X-ray source	PAL-5C beam line
Space group	*P*2_1_
Unit-cell parameters (Å, °)	a = 49.4, b = 76.6, c = 64.7, α = γ = 90, and β = 105.3
Wavelength (Å)	0.9796
Resolution (Å)	50.00–2.32 (2.36–2.32)
Total reflections	69,131
Unique reflections	19,691 (911)
Average I/σ (I)	42.7 (22.4)
*R*_merge_^a^	0.067 (0.165)
Redundancy	3.5 (3.5)
Completeness (%)	97.0 (90.2)
Refinement	
Resolution range (Å)	33.81–2.32 (2.38–2.32)
No. of reflections of working set	18,706 (1341)
No. of reflections of test set	964 (47)
No. of amino acid residues	554
No. of water molecules	208
*R*_cryst_^b^	0.176 (0.165)
*R*_free_^c^	0.235 (0.239)
R.m.s. bond length (Å)	0.0096
R.m.s. bond length (°)	1.596
Average B value (Å^2^) (protein)	27.9
Average B value (Å^2^) (solvent)	31.0

Values in parentheses refer to the highest resolution shells

^a^ $$ R_{\text{merge}} = \sum {{{\left| {\left\langle {\text{I}} \right\rangle - {\text{I}}} \right|} \mathord{\left/ {\vphantom {{\left| {\left\langle {\text{I}} \right\rangle - {\text{I}}} \right|} {\sum {\left\langle {\text{I}} \right\rangle } }}} \right. \kern-0pt} {\sum {\left\langle {\text{I}} \right\rangle } }}} $$

^b^ $$ R_{\text{cryst}} = \sum {{{\left| {\left| {\text{Fo}} \right| - \left| {\text{Fc}} \right|} \right|} \mathord{\left/ {\vphantom {{\left| {\left| {\text{Fo}} \right| - \left| {\text{Fc}} \right|} \right|} {\sum {\left| {\text{Fo}} \right|} }}} \right. \kern-0pt} {\sum {\left| {\text{Fo}} \right|} }}} $$

^c^*R*_free_ calculated with 5% of all reflections excluded from the refinement stages using high-resolution data

### Structure determination

The *Sf*SFGH crystal belongs to the monoclinic space group of *P*2_1_, with unit cell parameters of a = 49.4, b = 76.6, c = 64.7 Å, α = γ = 90, and β = 105.3°. The crystal structure in *Sf*SFGH was solved by a molecular replacement method using the *MOLREP* program from the *CCP4i* package [27, 28]. The crystal structure of esterase (OLEI01171) from *Oleispira Antarctica* (sequence identity 83%; PDB code 3i6y) was used as a template model [16]. The initial model was built iteratively and then refined using *Coot* and *REFMAC5* [29, 30]. The *phenix.refine* from *PHENIX* was also used for refinement [31]. The final model had an *R*_work_ of 17.6% and *R*_free_ of 23.5%. *Molprobity* was used for quality validation of the final model [32]. The detailed refinement statistics are provided in Table 3. The atomic coordinates and experimental structure factors of *Sf*SFGH were deposited in the RCSB Protein Data Bank under accession code 6JZL. All graphical representations of protein structures were prepared using *PyMOL* [33].

### Mass spectrometry and analytical ultracentrifugation

The molecular weight of *Sf*SFGH-His_6_ was determined by matrix-assisted laser desorption/ionization time of flight mass spectrometry (MALDI-TOF) using Voyager DE STR (Applied Biosystems, NCIRF, Seoul, Korea). To investigate the oligomeric state of *Sf*SFGH in solution, analytical ultracentrifugation (AUC) was performed using a ProteomeLab XL-A (Beckman Coulter, Brea, CA, USA), in buffer conditions of 150 mM NaCl and 20 mM Tris–HCl pH 8.0 at 20 °C. The sample was centrifuged at 40,000 rpm for 10 min and the sedimentation profile was monitored at a wavelength of 280 nm. Data were analyzed using the program SEDFIT [34, 35].

### Enzymatic assays

In general, the carboxyl esterase activity of *Sf*SFGH was assessed against *p*-nitrophenyl esters by measuring absorbance at 405 nm using an EPOCH2 microplate reader (Biotek). The standard assay solution consisted of 250 µM substrate solution in 100 mM NaCl and 20 mM Tris–HCl pH 8.0 with 10 µg *Sf*SFGH protein (at a final concentration of ~ 1.03 µM). The substrate specificity of *Sf*SFGH was determined using *p*-nitrophenyl esters varying in acyl-chain length, e.g., *p*-nitrophenyl phosphate (*p*-NP), *p*-nitrophenyl acetate (*p*-NA), butyrate (*p*-NB), hexanoate (*p*-NH), octanoate (*p*-NO), decanoate (*p*-ND), and dodecanoate (*p*-NDD).

The optimum pH of *Sf*SFGH was determined using *p*-NA with buffers at different pH. Enzymatic activities that differed by pH were measured after incubating the enzyme for 1 h at various pH values ranging from 3.0 to 10.0. The thermal stability of *Sf*SFGH was examined using *p*-NA. Activity changes by temperature were measured after incubating the enzyme at 25 °C, 37 °C, 50 °C, 60 °C, and 70 °C for 1 h. Aliquots were taken every 15 min to measure the residual activity of *Sf*SFGH at each given temperature. To assess effect of low temperature on the activity of *Sf*SFGH, activity was studied by equilibrating the enzyme at 4 °C. The activity was measured using *p*-NA as a substrate. The chemical stability of *Sf*SFGH was determined using *p*-NA with buffers containing diverse chemicals. The residual activities of *Sf*SFGH were measured after incubating the enzyme for 1 h in diverse conditions, such as 10% and 30% ethanol (EtOH), 30% isopropanol (I-PrOH), 0.1% sodium dodecyl sulfate (SDS), urea (from 0 M to 5 M), 1% Triton X-100 (Tx-100), 1% Tween 20 (Tw20), and 5 mM phenylmethylsulfonyl fluoride (PMSF). Measurement of enzymatic activity was performed in the same manner as the esterase activity assay described above.

### Thermal and chemical unfolding

The thermal unfolding of *Sf*SFGH was assessed using Far-UV CD measurements on a J-715 spectropolarimeter (JASCO) equipped with a thermostat-containing cell holder. The CD signal was monitored at 222 nm from 20 to 90 °C at a rate of 1.0 °C min^−1^. Chemical unfolding of *Sf*SFGH was induced by incubating the protein with increasing concentrations of urea (from 0 to 5 M) at room temperature for 1 h. Fluorescence measurements were performed using a FP-6200 spectrofluorometer (JASCO) in which the samples were excited at 280 nm and emission spectra were recorded in the range of 300 to 400 nm. All fluorescence spectra were recorded at a scan speed of 250 nm min^−1^ with a 5-nm slit width.

### Determination of kinetic parameters

Kinetic assays were performed using *p*-NA, *p*-NB, and *p*-NO as substrates. Enzyme solutions were prepared in 120 mM NaCl and 20 mM Tris–HCl pH 8.0 buffer. Various concentrations of substrate were added to the enzyme solutions, and the reactions were performed at RT. The absorbance changes in the reaction mixtures were monitored using an EPOCH2 microplate reader (Biotek) at 405 nm. Measurements were taken along with a blank, every 10 s for 2 min, and at least three measurements were collected to determine the rate at each substrate concentration. The molar extinction coefficients of *p*-nitrophenol and hydrolyzed nitrocefin were determined experimentally (*p*-nitrophenol; 17,154 M^−1^ cm^−1^). Reaction velocities were determined from the initial linear portion of progress curves, which were generated after subtracting the absorbances of the blank experiments, correcting sample path-length, and converting the absorbance to product concentration [P] with the experimentally obtained extinction coefficient. The kinetic parameters (*V*_max_ and *K*_m_) of *Sf*SFGH were obtained from the x- and y-intercepts of the Lineweaver–Burk plot. The *k*_cat_ was calculated using the equation $k_{\text{cat}} = {{V_{\text{max} } } \mathord{\left/ {\vphantom {{V_{\hbox{max} } } {\left[ {\text{E}} \right]}}} \right. \kern-0pt} {\left[ {\text{E}} \right]}}_{\text{total}}$.

### Bioinformatics analysis

For functional classification and sequence analysis of *Sf*SFGH, proteins related to *Sf*SFGH were identified using a BLAST search on the Protein Data Bank (PDB). All primary sequences were retrieved from the NCBI database in FASTA format. Multiple sequence alignments were performed using Clustal Omega [36] with the enzymes found using BLAST, and the results were rendered using ESPript [37]. For gene cluster analysis, homologous proteins of *Sf*SFGH (sequence identity: ≥ 80%) were collected from a BLAST search on the non-redundant protein sequence database, followed by retrieval of chromosome information for the collected proteins. The gene locus encoding *Sf*SFGH and its surroundings in the chromosome of *Shewanella frigidimarina* were compared with the corresponding regions in other *Shewanella* species, including *S. sediminis*, *S. woodyi*, *S. pealeana*, *and S. piezotolerans*.

### Site-directed mutagenesis

To obtain mutants of *Sf*SFGH, mutagenesis was carried out using the Quik-Change site-directed mutagenesis method [19]. Chemically synthesized oligonucleotides were used to substitute each residue (purchased from Cosmogenetech, Korea). In the Quik-Change method, the PCR mixture contained ~ 300 ng DNA template, 4 μL 5× Pfu DNA polymerase master mix, and 1 μM of each primer in a total volume of 20 μL. The mixture was heated at 95 °C for 2 min and then subjected to thermal cycling (18 cycles at 95 °C for 1 min, 66 °C for 1 min, and 72 °C for 7 min). The PCR product was incubated with DpnI at 37 °C for 1 h, subsequently incubated for 10 min at 80 °C, and transformed into *E. coli* DH5α. Three colonies were randomly selected for sequencing. The verified plasmid was then transformed into *E. coli* BL21(DE3) for overexpression.

## Additional file

**Additional file 1: Fig. S1.** Phylogenetic analysis of *Sf*SFGH. **Fig. S2.** Gene clustering analysis of *Sf*SFGH. **Fig. S3.** Recombinant *Sf*SFGH protein purification, crystallization, and X-ray diffraction data collection. Fig. S4. Freeze–thaw cycles of *Sf*SFGH.
