# Supplementary material for: Structural and functional characterization of a novel cold-active S-formylglutathione hydrolase (SfSFGH) homolog from Shewanella frigidimarina, a psychrophilic bacterium
Source: Microb Cell Fact. 2019 Aug 19;18:140. doi: 10.1186/s12934-019-1190-1 (PMC6699074; doi:10.1186/s12934-019-1190-1)
Supplement: Supplementary file 1 — Additional file 1: Fig. S1. Phylogenetic analysis of SfSFGH. Fig. S2. Gene clustering analysis of SfSFGH. Fig. S3. Recombinant SfSFGH protein purification, crystallization, and X-ray diffraction data collection. Fig. S4. Freeze–thaw cycles of SfSFGH. [file 12934_2019_1190_MOESM1_ESM.docx]

**Fig. S1** Phylogenetic analysis of *Sf*SFGH. PSI-BLAST was used to retrieve sequences similar to *Sf*SFGH [1]. Sequence alignment with retrieved sequences was conducted with Clustal Omega [2], and the result was rendered using ESPript [ref]. To identify and classify *Sf*SFGH, representative sequences from each esterase family were aligned and a phylogenetic tree was constructed in Molecular Evolutionary Genetics Analysis (MEGA) v.7.0 by the neighbor-joining method with 5,000 iterations [3].

1. Altschul SF, Madden TL, Schäffer AA, Zhang J, Zhang Z, Miller W, Lipman DJ: **Gapped BLAST and PSI-BLAST: a new generation of protein database search programs.** *Nucleic acids research* 1997, **25:**3389-3402.

2. Sievers F, Wilm A, Dineen D, Gibson TJ, Karplus K, Li W, Lopez R, McWilliam H, Remmert M, Söding J: **Fast, scalable generation of high‐quality protein multiple sequence alignments using Clustal Omega.** *Molecular systems biology* 2011, **7:**539.

3. Kumar S, Stecher G, Tamura K: **MEGA7: molecular evolutionary genetics analysis version 7.0 for bigger datasets.** *Molecular biology and evolution* 2016, **33:**1870-1874.


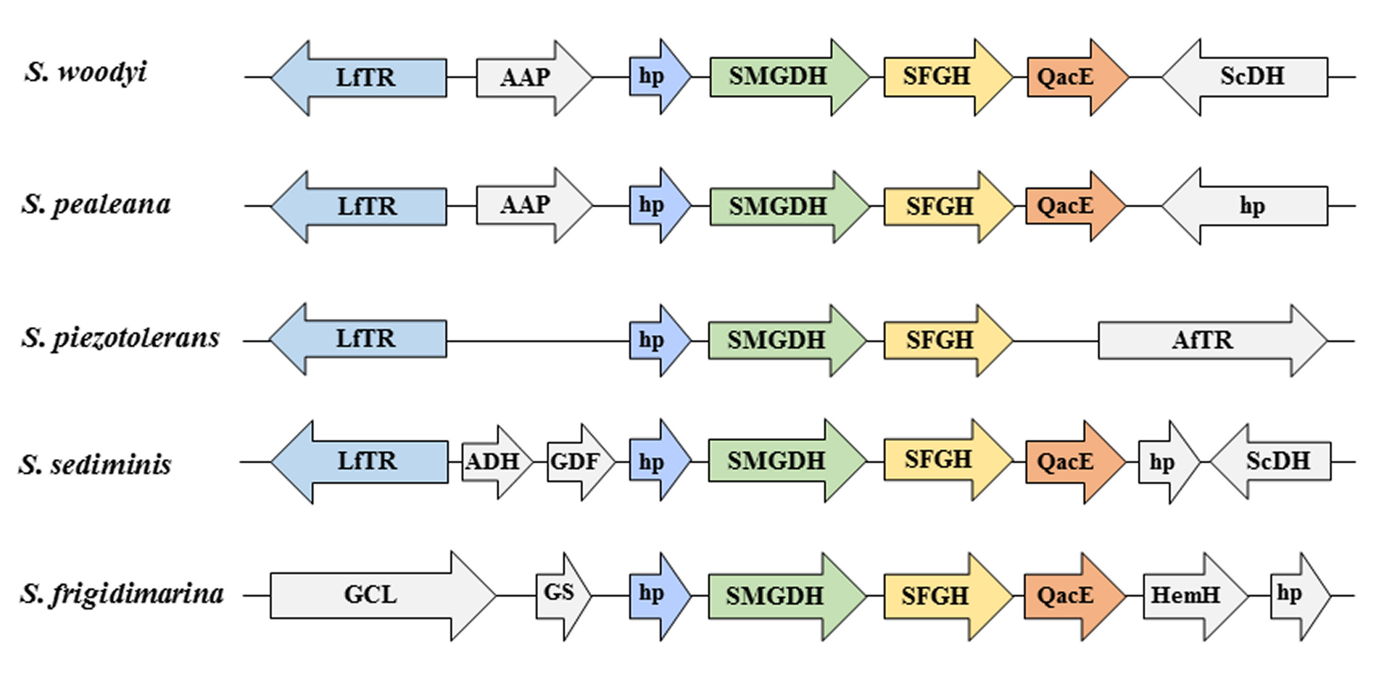


**Fig. S2** Gene clustering analysis of *Sf*SFGH. Highly conserved gene clusters surrounding SFGH gene were found in *S. woodyi, S. pealeana, S. piezotolerans, S. sediminis,* and *S. frigidimarina*. LfTR: LysR family transcriptional regulator; AAP: Aldehyde activating protein; hp: hypothetical protein; SMGDH: S-(hydroxymethyl)glutathione dehydrogenase; SFGH: *S*-formylglutathione hydrolase; QacE: QacE SMR transporter; ScDH: short-chain dehydrogenase; AfTR: AraC family transcriptional regulator; GCL: Glutathione-cysteine ligase; HemH: ferrochelatase.

**Fig. S3** Recombinant *Sf*SFGH protein purification, crystallization, and X-ray diffraction data collection. (A) Purified *Sf*SFGH protein (1 μg) was loaded and visualized by 15% SDS-PAGE. Lane M indicates the protein molecular-weight markers (kDa). (B) Analytical ultracentrifugation (AUC) experiments using 0.5 mg/ml *Sf*SFGH revealed a mass of 62.3 kDa (sedimentation coefficient, 4.209 S; frictional ratio, 1.34), indicating that *Sf*AcE is a stable dimer in solution. The calculated molecular weight of the monomer *Sf*SFGH is 29.7 kDa based on the amino acid sequence. (C) Morphology of *Sf*SFGH crystals grown for 7 days using 0.2 M lithium chloride and 16% (w/v) PEG 3350; these crystals were subsequently used for X-ray diffraction data collection. (D) A representative X-ray diffraction image was obtained from the *Sf*SFGH crystal with a maximum resolution limit of 2.32 Å.


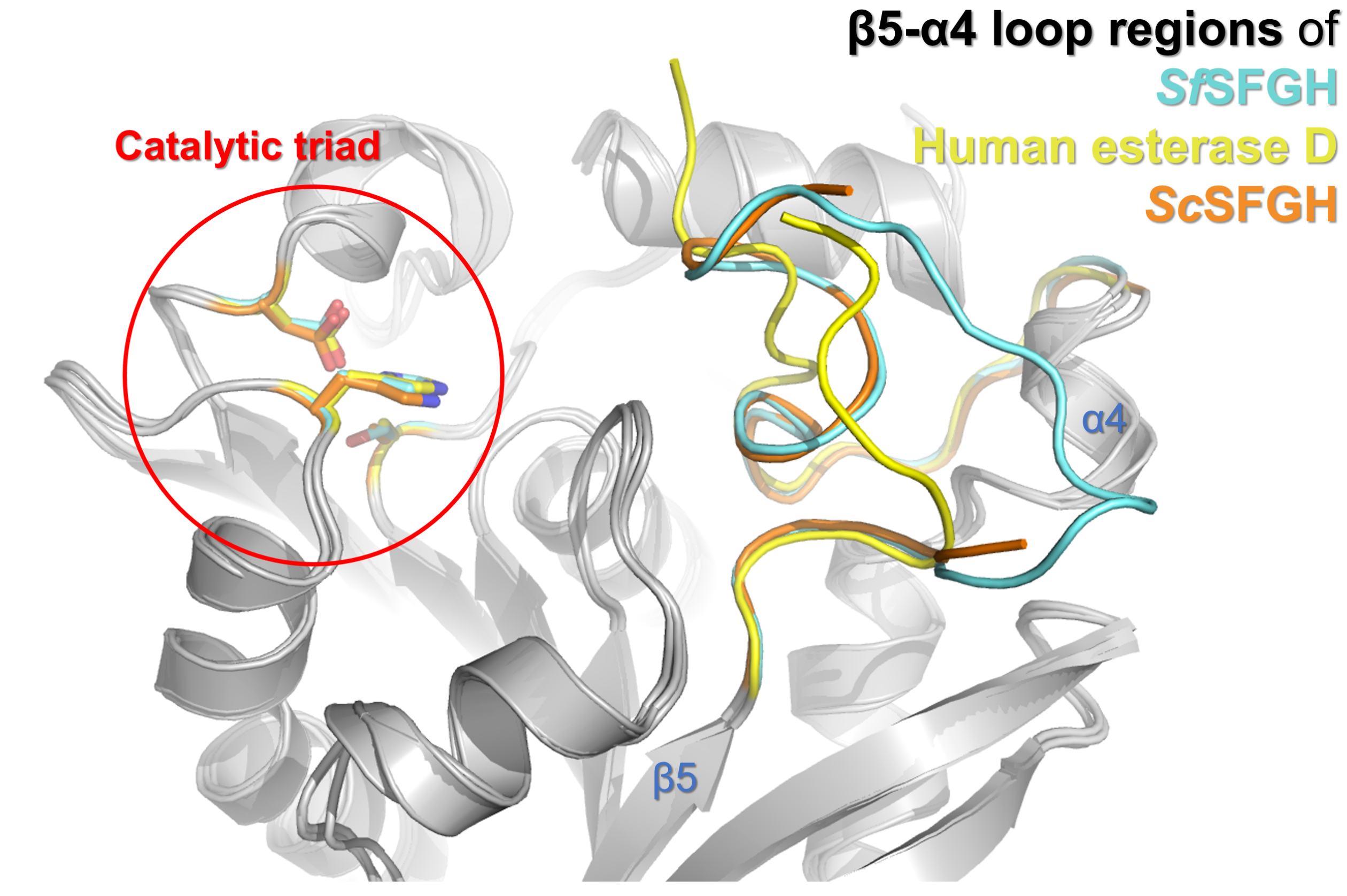


**Fig. S4** *Sf*SFGH has more opened active site compared with those of mesophilic homologs. Structural comparisons of *Sf*SFGH (aquamarine color) with human esterase D (PDB code 3FCX: yellow color) and *Saccharomyces cerevisiae* SFGH (*Sc*SFGH: PDB code 4FLM: orange color) show that even if architecture of catalytic triad region is highly conserved, the β5-α4 loop region of *Sf*SFGH has more open conformation.

**Fig. S5** Freeze-thaw cycles of *Sf*SFGH. Residual activity of *Sf*SFGH was monitored after repeated freezing and thawing cycles. *Sf*SFGH at 1 mg/ml was frozen at -80 °C for 5 min and then thawed at room temperature for 10 min. From each cycle, 10 μg *Sf*SFGH was collected and incubated with 250 μM *p*-nitrophenyl acetate. Absorbance was measured at 405 nm.
